# Forearm mystery: rare flexor carpi radialis muscle cysticercosis

**DOI:** 10.1093/jscr/rjae754

**Published:** 2024-12-15

**Authors:** Mohd Fahud Khurram, Nimisha Singh

**Affiliations:** Department of Plastic and Reconstructive Surgery, Jawaharlal Nehru Medical College, Aligarh Muslim University, Aligarh, Uttar Pradesh 202001, India; Department of Plastic and Reconstructive Surgery, Jawaharlal Nehru Medical College, Aligarh Muslim University, Aligarh, Uttar Pradesh 202001, India

**Keywords:** Myocystecercosis, Tenia solium, albendazole

## Abstract

This case report describes a rare instance of muscle cysticercosis in a 16-year-old vegetarian female from an endemic region, highlighting the challenges in diagnosing atypical presentations of the disease. The patient presented with a 2-month history of persistent pain and swelling in the right forearm, which did not respond to over-the-counter analgesics. A clinical examination identified a non-tender, immobile swelling, and imaging studies suggested cysticercosis. Despite treatment with albendazole and non steroidal anti-inflammatory drugs (NSAIDs), the swelling persisted, necessitating surgical intervention. The excised cyst was confirmed as cysticercosis through histopathological examination. This case underscores the importance of considering cysticercosis in the differential diagnosis of soft tissue swellings, particularly in endemic areas, and emphasizes the role of imaging and histopathology in ensuring accurate diagnosis. Preventive measures such as improved sanitation and hygiene are crucial in controlling the spread of cysticercosis.

## Introduction

Human cysticercosis is commonly found across various regions, such as Africa, Southeast Asia, and Eastern Europe [1]. The prevalence is significantly greater in impoverished regions as a result of a convergence of factors including rural living conditions, high population density, and insufficient sanitation. These circumstances promote greater interaction between humans and pigs, hence increasing the likelihood of faecal contamination. Cysticercosis is highly prevalent in Northern India, specifically in the states of Bihar, Uttar Pradesh, and Punjab [2].

Neurocysticercosis is a disease resulting from the larvae of the tapeworm *Taenia solium*, particularly the cysticercus cellulosae form [3]. Both humans and swine serve as hosts for this parasite during its lifecycle. The adult parasites are contained in the small intestines of humans, who are the primary hosts. Pigs are the secondary hosts, harbouring the larval stages [4]. Pigs in regions with inadequate sanitation consume eggs that are produced in the excrement of taenia-infected humans. Eggs are absorbed into the bloodstream after they are ingested and pass through the intestinal wall. In the tissues of the intermediate host, such as the striated muscles, they develop into oncospheres and subsequently into metacestodes. These embryonic forms, known as metacestodes, develop into cysticerci, which are vesicular structures that contain a scolex that is folded inward. Humans consume undercooked pork that contains cysticerci, which causes the larvae to enter the gastrointestinal tract, protrude, attach to the mucosa, and mature into adult worms. These mature worms subsequently produce eggs, which are ejected through human excrement, thereby maintaining the life cycle. The human host of the tapeworm is a source of infection for both humans and swine [5]. The human gut (Taeniasis), brain (neurocysticercosis, NCC), or musculature (cysticercosis) are where the parasites can be found, whether in their mature form or as larvae [6]. The mature *T. solium* exhibits a remarkable capacity for fecundity and reproduction, ensuring the species’ survival and longevity [7]. Because of this, it is imperative to concentrate on the individual who possesses the human tapeworm in order to implement control measures. In this report, we describe an unusual case of muscle cysticercosis, marked by persistent forearm swelling that remained despite conservative management.

## Case report

**Figure 1:**
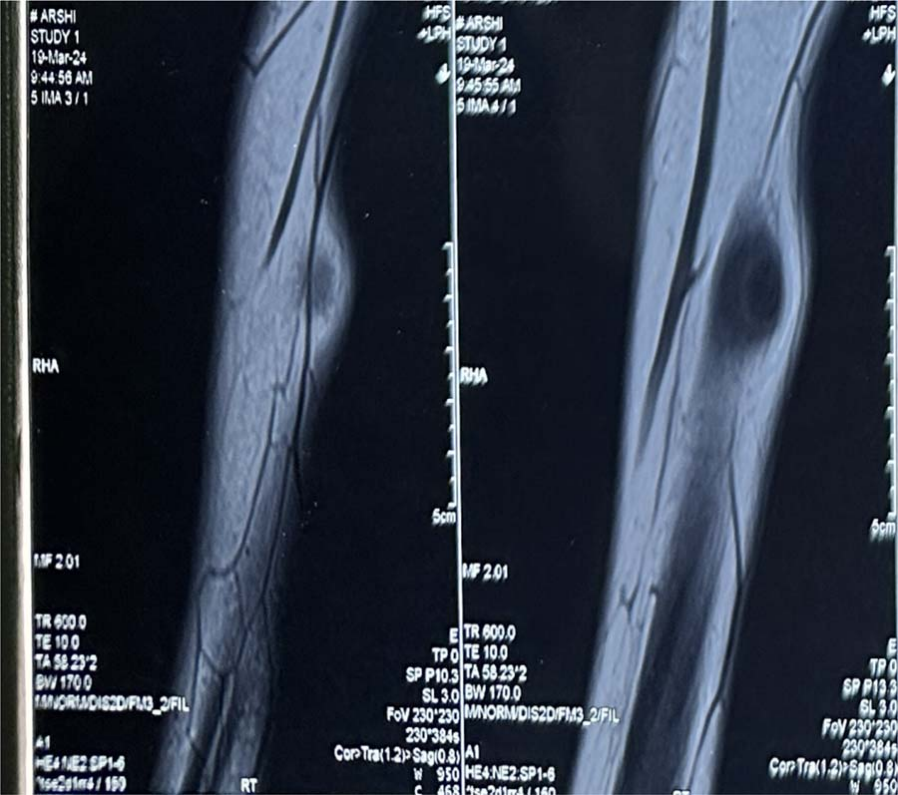
MRI Soft tissue of right forearm: suggestive of well-defined cystic lesion with eccentric mural nodule in intramuscular plane with mild perilesional oedema.

**Figure 2:**
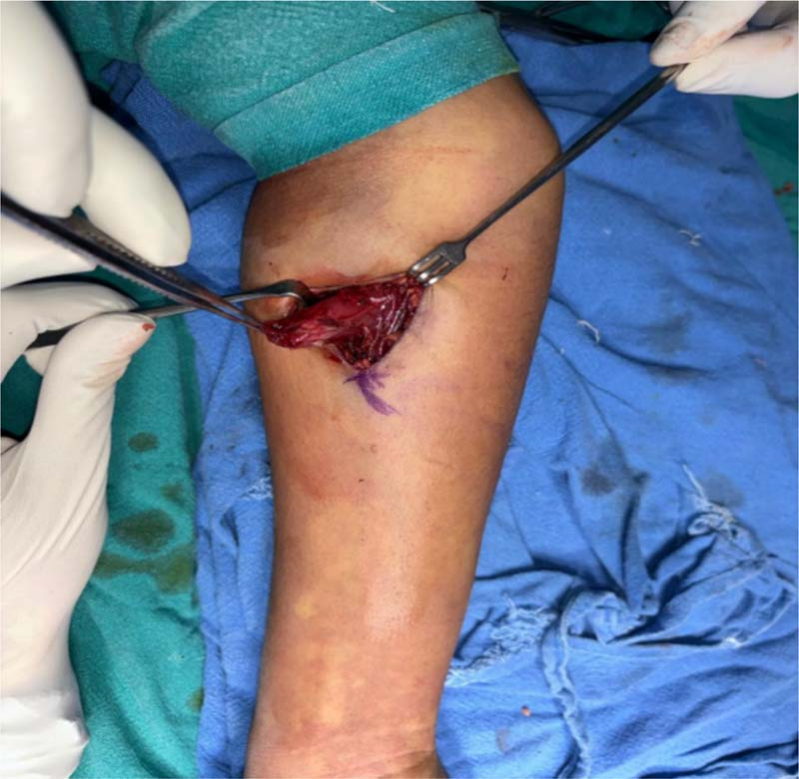
Embedded swelling with in flexor carpi radialis muscle.

**Figure 3:**
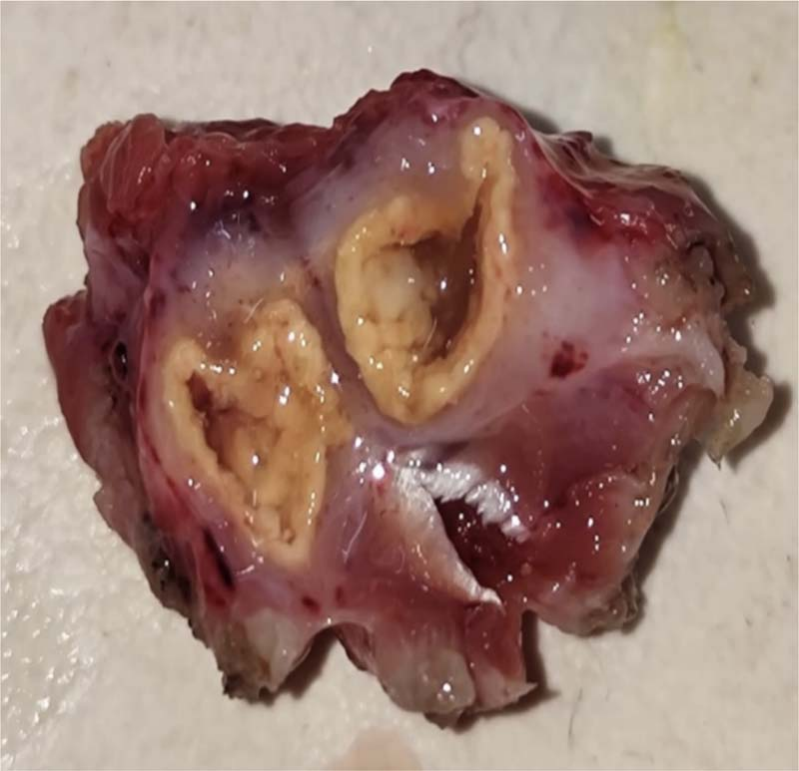
On table, cut section of the specimen showing calcified wall.

**Figure 4:**
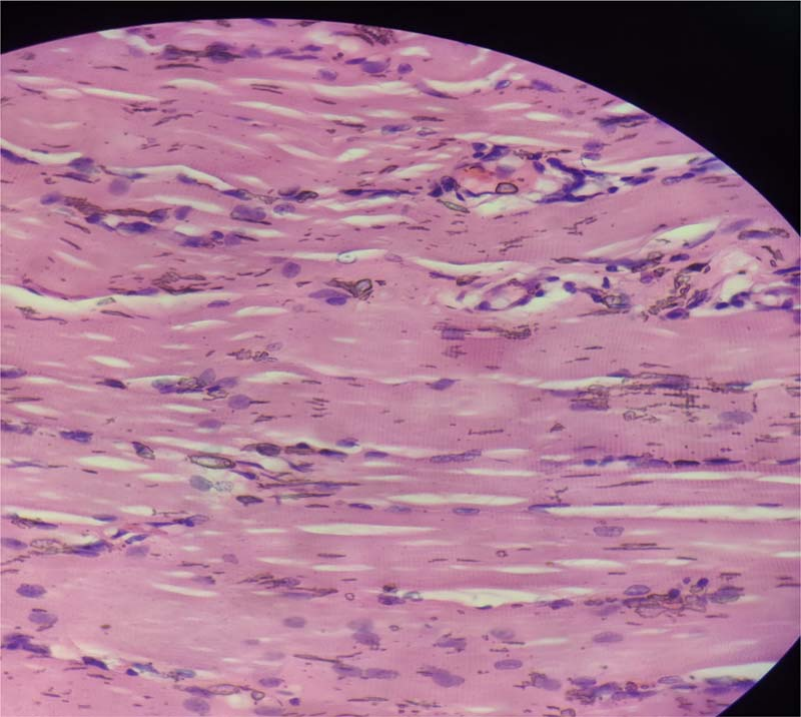
Haematoxylin and eosin stained section showing skeletal muscle fibres with chronic inflammatory infiltrate.

A 16-year-old female student who adheres to a vegetarian diet and has no known comorbidities presented to the Plastic Surgery outpatient department with a 2-month history of pain and swelling in the right forearm. There was no history of headache, seizure, or blurred vision. Despite the administration of over-the-counter analgesics, she did not experience any relief. In the proximal third of the right forearm, she was found to have swelling measuring 3 × 3 cm during the examination. It was non-tender, immobile, and attached to the underlying muscle, with a firm to rigid consistency. She was referred to the radiologist for a soft tissue ultrasound, which revealed a 14.6 × 4 mm hypoechoic lesion in the flexor aspect of the proximal right forearm. The lesion was accompanied by moving linear internal echoes, which are indicative of worm infestation/cysticercosis. The soft tissue MRI was indicative of a well-defined cystic lesion with an eccentric mural nodule in the intramuscular plane, as well as mild perilesional oedema (Fig. 1). The patient received oral Albendazole at a dose of 400 mg twice daily combined with nonsteroidal anti-inflammatory medications for 6 weeks; however, the swelling did not improve with this treatment. She was subsequently admitted and planned for surgical excision. Biochemical tests yielded normal results, and after obtaining informed consent, she proceeded to surgery. The cyst was discovered intraoperatively, embedded between the muscle fibres (Fig. 2). It was excised on the table, revealing scolex (Fig. 3), and the cut specimen was subsequently sent for histopathological examination which confirmed the diagnosis (Fig. 4). Albendazole was also recommended to the patient’s family members in order to prevent recurrence and eliminate the carrier state. At the 2-month follow-up, the patient is asymptomatic and conducting all of her activities in the same manner as before.

## Discussion

The significance of taking into account uncommon manifestations of cysticercosis, such as muscle involvement, is highlighted by the case studied. This is especially true in regions where the disease is prevalent. The most prevalent presentation is neurocysticercosis; however, extra neuronal involvement presents diagnostic challenges and necessitates a multidisciplinary approach for precise diagnosis and management. A comprehensive evaluation was initiated after the patient’s initial clinical presentation of isolated forearm swelling in order to exclude a variety of differential diagnoses, such as soft tissue tumours, abscesses, or inflammatory conditions. In 25% of cysticercosis cases, teniasis is associated. Prior to diagnoses of isolated cysticercosis, it is imperative to exclude this possibility [8]. The diagnostic accuracy of cysticercosis was significantly altered by the introduction of computed tomography (CT) and magnetic resonance imaging (MRI), as they offer confirmation of the number and topography of lesions, as well as their stage of involution [9]. Enzyme-linked immunoelectron transfer blot assays can be employed to identify antibodies to species-specific antigens of *T. solium* [10]. The laboratory findings of eosinophilia, increased IgG levels, and, most importantly, a positive enzyme-linked immunosorbent assay (ELISA) test for IgG antibodies against *T. solium* are crucial indicators [11]. Promoting the inspection of green leafy vegetables and meat, thoroughly washing produce, cooking meat completely, consuming boiled or filtered water, and washing hands thoroughly before meals should be practiced [12]. In addition, the prevention and management of cysticercosis in endemic regions are contingent upon the enhancement of public health measures, such as sanitation and hygiene practices.

## Conclusion

In endemic regions, the case report of isolated muscle cysticercosis in the forearm emphasizes the necessity of being cognizant of uncommon cysticercosis presentations. This case serves as an example of the diagnostic obstacles that atypical manifestations present, necessitating a comprehensive assessment and a multidisciplinary approach to ensure exact diagnosis and treatment. Reducing the prevalence of cysticercosis necessitates preventive measures, such as educating the public on appropriate food handling and cooking practices, promoting hygiene, and enhancing sanitation. It is possible to achieve favourable outcomes and prevent complications by implementing early diagnosis and appropriate treatment.
